# Low-Dose Taxol Promotes Neuronal Axons Extension and Functional Recovery after Spinal Cord Injury

**DOI:** 10.1155/2023/5604103

**Published:** 2023-01-27

**Authors:** Baoguo Liu, Sumei Liu, Dejun Sun

**Affiliations:** ^1^College of Pharmacy, Jilin University, Changchun 130021, China; ^2^Xuanwu Hospital Capital Medical University, Beijing 100053, China

## Abstract

Axonal regeneration has been the research focus in the field of clinical treatment for spinal cord injury (SCI). The growth and extension of neuronal axons is a dynamic biological process mediated by the cytoskeleton, and microtubule plays an important role in axonal growth. Moderate stabilization of microtubule promotes axonal growth and eliminates various intra- and extracellular mechanisms that impede axonal regeneration. After SCI, the damaged axons rapidly form a growth cone, wherein the stability of tubulin decreases, impairing axonal regeneration. Taxol with proven clinical safety is commonly used as a broad-spectrum antitumor drug. Importantly, Taxol can promote axonal extension by enhancing and stabilizing the microtubule assembly. In our study, we systematically investigated the differentiation of neural stem cells (NSCs) *in vitro* and functional recovery in injured rats *in vivo* following Taxol treatment. Low-dose Taxol promoted differentiation of NSCs to neurons and significantly extended the axons *in vitro*. *In vivo*, Taxol promoted the expression of *β*III-tubulin in the injured areas and motor function recovery after SCI. Low-dose Taxol is a promising clinical agent to promote axonal regeneration after SCI.

## 1. Introduction

Axonal regeneration is a research hotspot in studies on regenerative therapy for spinal cord injury (SCI) [1]. The growth and extension of axons is a cytoskeleton-mediated biological process. The dynamic assembly of the cytoskeleton and its transport function is the molecular basis of axon growth [2]. Actin filaments and microtubules comprise the main cytoskeleton framework of neurons, and the latter plays an important role in axonal growth [3]. Axonal growth occurs mainly within the growth cone, wherein the regulation of the microtubule dynamics is crucial [4].

Microtubules guide the axonal extension and retraction by polymerization and depolymerization. After SCI, axon microtubules are depolymerized, making it difficult to provide the necessary pressure for the outward growth of axons and the damaged axons rapidly form growth cones, whereby the stability of microtubules is related to axonal regeneration after nerve injury [5]. Moderate stabilization of microtubule prevents the axonal retraction and enlargement of apical axons after injury, thus overcoming the growth inhibitory effects of myelin in the central nervous system [6]. The moderate stabilization of microtubules also eliminates various intracellular mechanisms that impair axonal regeneration. Taxol, a microtubule stabilizer, increases the level of tubulin and results in tight binding of microtubules to Kinesin-1, thereby altering intracellular transport of Kinesin-1 and Dynein and promoting microtubule polymerization [7, 8]. Taxol is commonly used as a broad-spectrum antitumor drug, and its clinical safety has been confirmed. Currently, Taxol is the only drug that promotes and stabilizes microtubule assembly. In recent years, studies have reported that Taxol can promote axonal extension by stabilizing microtubules, which has drawn the attention of many researchers as it provides a new perspective for assessing axonal regeneration after SCI. However, the application of Taxol in promoting axonal regeneration after SCI remains in the preliminary stages.

Herein, we investigated the roles of Taxol on neural stem cells (NSCs) differentiation into neurons and axonal elongation *in vitro* and *in vivo*. Taxol promoted neuronal differentiation from NSCs and axonal elongation, making it promising for the clinical treatment of SCI in the future.

## 2. Materials and Methods

### 2.1. Culture of Primary Brain-Derived Neural Stem Cells

All of the animals were purchased from Beijing Vital River Laboratory Animal Technology Co., Ltd., Beijing, China. Brain-derived neural stem cells (BNSCs) from neonatal SD rats within 24 hours were isolated according to a previously reported protocol [9–12] and the experiments were approved by the ethics committee of Xuanwu Hospital Capital Medical University. The neural stem cell (NSC) proliferation medium comprising DMEM/F12 (Invitrogen) + N2 (Invitrogen) + B27 (Invitrogen) + GlutaMAX (Gibco) + NEAA (Gibco) + Penicillin-Streptomycin (Gibco) +20 ng/mL bFGF (PeproTech) +20 ng/mL EGF (PeproTech) was daily replaced by fresh medium. The primary BNSCs were cultured for 7 days.

### 2.2. Identification and Differentiation of BNSCs

The BNSCs spheres with diameters of approximately 100-150 *μ*m were dissociated into single cells, 5 × 10^4^ cells/well were seeded evenly on a circular PDL/Laminin-coated coverslip of 12 mm diameter, and the NSC adherent culture medium comprising DMEM (Invitrogen) +10% FBS (Invitrogen) + GlutaMAX (Gibco) + NEAA (Gibco) + Penicillin-Streptomycin (Gibco) was added. Subsequently, the cells were grown at 37°C in a CO_2_ incubator for 24 hours and allowed to adhere. The cells were fixed with 4% paraformaldehyde and stained for immunofluorescence assays with NSC-specific antibodies against Nestin (1 : 500, Sigma) and Vimentin (1 : 500, Sigma).

Differentiation capacity was assessed as follows. BNSCs spheres were dissociated into single cells using 0.25% Trypsin (Invitrogen) at 37°C. After 24-hour adherent cell culture as described above, the NSC differentiation medium comprising DMEM/F12 (Invitrogen) + N2 (Invitrogen) + B27 (Invitrogen) + GlutaMAX (Gibco) + NEAA (Gibco) + Penicillin-Streptomycin (Gibco) was added, and the cells were allowed to differentiate at 37°C in a 5% CO_2_ incubator for 7 days. Half the amount of NSC differentiation medium was changed every 2 days. After 7 days, then the cells were fixed with 4% paraformaldehyde. Subsequently, these were stained for immunofluorescence assays using the neuron-specific antibody, anti-Tuj1 (*β*III-tubulin, 1 : 500, Sigma), and the glial cell-specific antibody, anti-GFAP (1 : 800, Sigma).

### 2.3. Cytotoxicity Assay for Taxol

The Cell Counting Kit-8 (CCK-8, Dojindo Beijing Co., Ltd) was used to determine the cytotoxicity of Taxol following the manufacturer's instructions. The BNSCs spheres were dissociated into single cells, and 1 × 10^4^ cells/well were seeded evenly into a PDL/Laminin-coated 96-well plate. Taxol at concentrations of 0 nmol/L, 1 nmol/L, 2 nmol/L, 3.5 nmol/L, 5 nmol/L, 7 nmol/L, 10 nmol/L, and 14 nmol/L, was added to the NSC differentiation medium in the eight groups, respectively. The cells were incubated in a 5% CO_2_ incubator at 37°C for 2 hours. The cytotoxicity assay was conducted following enzyme calibration with a 450 nm filter.

### 2.4. BNSCs Differentiation under Different Concentrations of Taxol

We selected six concentrations of Taxol and designed seven experimental groups as follows: 0 nmol/L (control), 1 nmol/L, 2 nmol/L, 3.5 nmol/L, 5 nmol/L, 7 nmol/L, and 10 nmol/L treatments. 5 × 10^4^ cells/well were seeded evenly on a circular PDL/Laminin-coated coverslip of 12 mm diameter, and the NSC adherent culture medium comprising DMEM (Invitrogen) +10% FBS (Invitrogen) + GlutaMAX (Gibco) + NEAA (Gibco) + Penicillin-Streptomycin (Gibco) was added. Cells were grown and allowed to adhere in a CO_2_ incubator at 37°C for 24 hours. Subsequently, the culture medium in the seven groups was replaced by the NSC differentiation medium with Taxol at different concentrations. The cells were then allowed to differentiate for 7 days at 37°C in a CO_2_ incubator. After 7 days, the immunofluorescence staining assay was performed using anti-Tuj1 (*β*III-tubulin, 1 : 500, Sigma), anti-Map2 (1 : 500, Sigma), and anti-GFAP (1 : 800, Sigma) antibodies. The cells were incubated with the primary antibodies overnight at 4°C followed by incubation with secondary antibodies at 26°C for 2 hours. The cells were scanned using a confocal microscope (Leica TCS SP5 CARS).

### 2.5. Taxol Treatment in the Rat Acute-Phase Spinal Cord Transection Model

Female SD rats weighing 200-220 g were selected as the experimental animals, and the rat T8-T9 spinal cord transection model was used for *in vivo* experiments. The experimental animals were purchased from Beijing Vital River Laboratory Animal Technology Co. Ltd., Beijing, China, and the animal experimental protocols were approved by the ethics committee of the Xuanwu Hospital Capital Medical University.

In this experiment, thrombin-fibrinogen materials with different doses of Taxol were immediately implanted into the lesion sites of rats with acute SCI. Thrombin-fibrinogen materials were obtained following the mixing of the same amount of human thrombin (final concentration 50 U/mL, solvent 0.1% BSA) and human fibrinogen (final concentration 50 mg/mL, solvent 20 mmol/L CaCl_2_). We injected Taxol into the thrombin-fibrinogen material and immediately implanted it into the SCI lesion site. The injured rats were assigned randomly into 4 groups according to the doses of Taxol in the transplant material as follows: 0.05 *μ*g/rat, 0.5 *μ*g/rat, 5 *μ*g/rat, and 0 *μ*g/rat treatments. There were 10 rats in each group. To facilitate a more accurate evaluation of the motor functions, 10 model rats with SCI were randomly selected as a reference baseline. All injured animals were raised in the Experimental Animal Barrier Environment at 26°C with 50% humidity and artificially assisted urination twice a day.

### 2.6. Functional Recovery Assay

We used a subjective Basso Beattie Bresnahan (BBB) score and an objective electrophysiological test to evaluate the recovery of motor functions of experimental animals. The BBB rating was performed for the rats after surgery in each group every 7 days in an open field with a diameter of 2 m [13–16]. A continuous 5-minute double-blind assessment of the motion characteristics of each experimental animal was performed.

On the 120th postoperative day, the cortical motor evoked potential (CMEP) and cortical somatosensory evoked potential (CSEP) of each rat was tested. The latency and amplitude of each group of rats were collected using the Keypoint EMG/evoked potential instruments and the Keypoint EMG/EP system was employed for data processing (Dandy Company, Denmark). The electrodes were placed according to the placement of the International 10-20 system electrodes. The stimulation intensity was 25 mV and 3.5 mA.

### 2.7. Tissue Sampling, Fixation, Dehydration, Sectioning, Staining and Observation

The rats were anesthetized. After perfusion, the spinal cord tissues were dissected and fixed in 4% paraformaldehyde for 72 hours, followed by immersion in 20% and 30% sucrose solutions for tissue dehydration, successively for 24 hours each. The frozen tissue was cut into 20 *μ*m thick sections (Leica CM1950).

Tissue sections were HE stained following the kit protocol (Beyotime C0105M). The images were captured using a digital slide scanner (3DHISTECH Pannoramic).

For the immunofluorescence assay, the tissue sections were treated with 0.3% PBST and blocked in 10% BSA for 1 hour. The sections were incubated with primary antibodies, including anti-GFAP (1 : 800, Sigma) and anti-Tuj1 (*β*III-tubulin, 1 : 500, Sigma) overnight at 4°C followed by secondary antibody incubation at 26°C for 2 hours. The tissue sections were scanned using a confocal microscope (Leica TCS SP5 CARS).

### 2.8. Statistical Analysis

10 magnification fields each at 200× were randomly selected per section for each group using the Leica Acquire software (n =5/group). The GraphPrism 7.0 software was used for quantitative data mapping and primary statistical analysis. The SPSS 27.0 statistical analysis software was used for all statistical analyses. *p* <0.05 indicated a statistically significant difference.

## 3. Results

### 3.1. BNSCs Expresses Neural Stem Cell Markers and Have the Ability to Differentiate into Neuronal Cells

**Figure 1:**
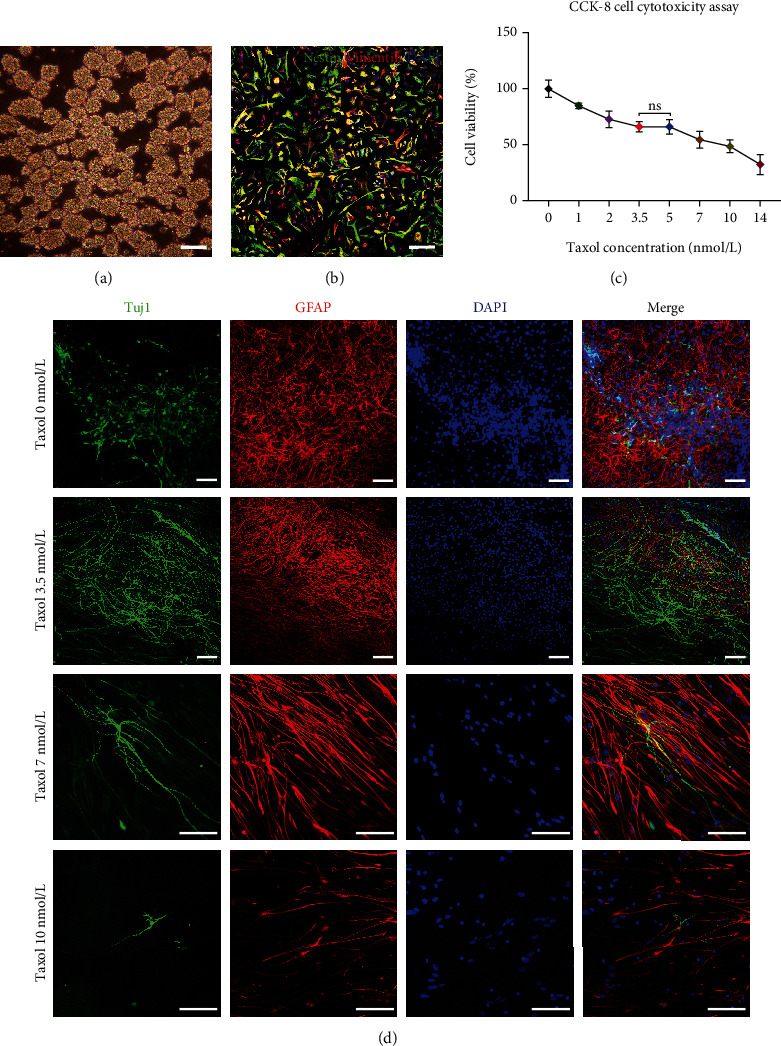
Identification and differentiation assay for BNSCs. (a) The BNSCs neurospheres are shown. (b) Immunofluorescence staining for BNSCs with antibodies against Nestin/Vimentin. (c) CCK-8 assay for detecting the cytotoxicity of Taxol on BNSCs. (d) BNSCs differentiation for 7 days under different concentrations of Taxol; Green, Tuj1; Red, GFAP; Blue, DAPI. Scale: 100 *μ*m.

We isolated the BNSCs *in vitro* (Figure 1(a)). Following anti-Nestin and anti-Vimentin staining, more than 95% of the BNSCs were found to be double-positive (Figure 1(b)). After 7 days of differentiation protocol, all BNSCs were found to differentiate into nerve cells with protrusions. Anti-Tuj1 and anti-GFAP staining were positive, indicating the ability of BNSCs to differentiate into neurons and glial cells. Taken together, we successfully obtained BNSCs.

### 3.2. Cytotoxicity Assay for Taxol at Different Concentrations

We tested the cytotoxic effects of Taxol on neuronal cells using the CCK-8 kit. A curve showing the continuous decrease in the survival rate with increasing Taxol concentration was obtained by calculating cell survival and inhibition rates. The survival rates of neuronal cells were all greater than 50% in the Taxol concentration range of 1-10 nmol/L. In the Taxol concentration range of 3.5-5 nmol/L, the survival rates of cells reached 66%, and the curve was horizontal (Figure 1(c)). Therefore, the survival rate of neural cells in the Taxol concentration range of 1-10 nmol/L was appropriate. We designed experiments with concentration revolving around the center range from 3.5-5 nmol/L to investigate the effects of Taxol on differentiation.

### 3.3. Taxol Promotes the Differentiation of NSCs Cultured *In Vitro* into Neurons with Long Axons

**Figure 2:**
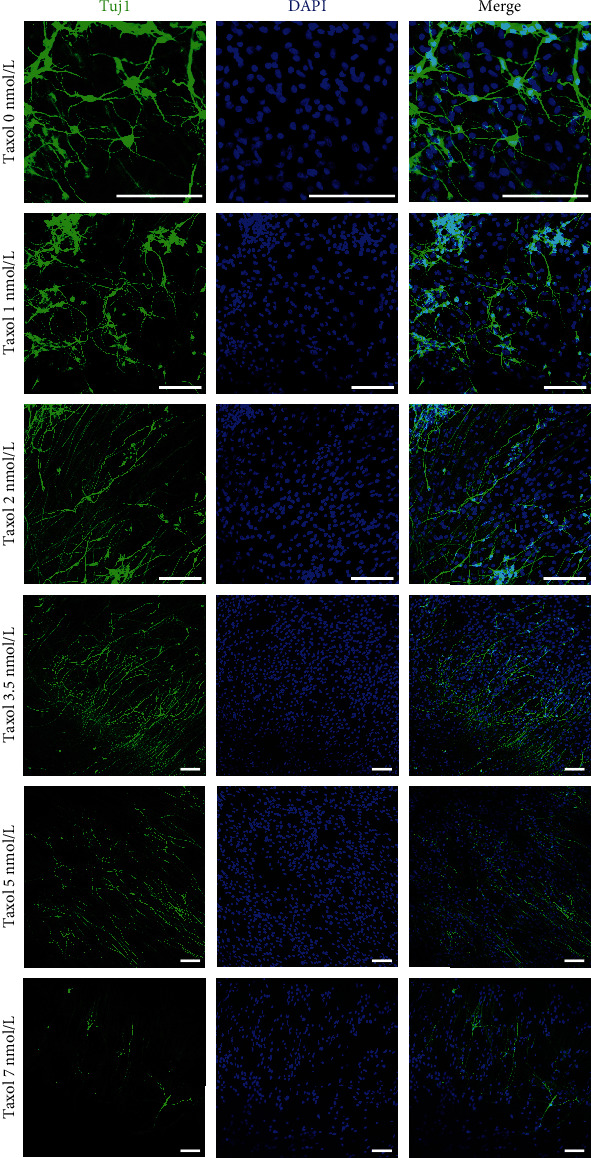
BNSCs differentiation under different concentrations of Taxol *in vitro*. Low-dose Taxol significantly increases the number of Tuj1-positive cells and the average axonal length of neurons is extended. Green, Tuj1; Blue, DAPI. Scale: 100 *μ*m.

**Figure 3:**
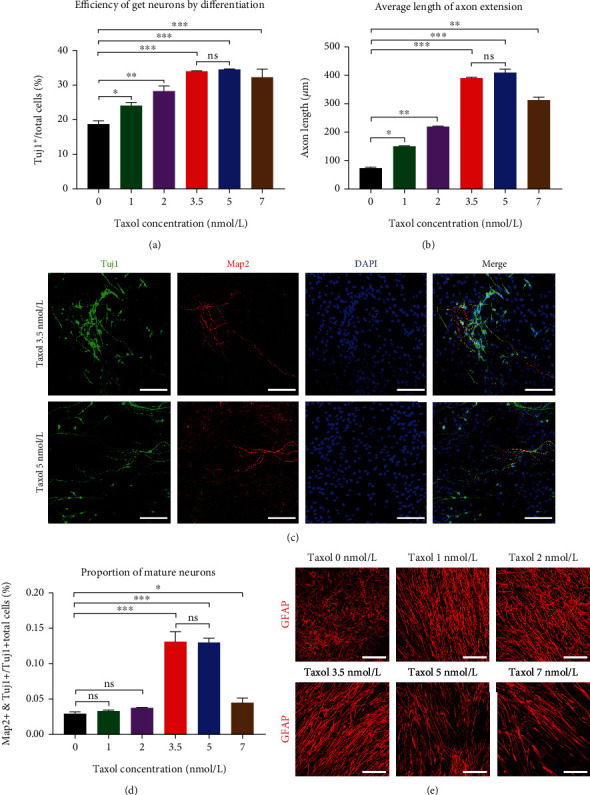
Low-dose Taxol promotes neuronal differentiation from BNSCs and extension *in vitro*. (a) Low-dose Taxol significantly increases the number of Tuj1-positive cells; ^∗^*p* <0.05, ^∗∗^*p* <0.01, ^∗∗∗^*p* <0.001. (b) Low-dose Taxol significantly extends the average axonal length of neurons, ^∗^*p* <0.05, ^∗∗^*p* <0.01, ^∗∗∗^*p* <0.001. (c) Effects of low-dose Taxol on BNSC differentiation *in vitro*; Green, Tuj1; Red, Map2; Blue, DAPI. Scale: 100 *μ*m. (d) During BNSCs differentiation, the addition of Taxol at a low dose increases the number of Tuj1 and Map2 positive co-stained cells. As compared to the no-Taxol group, Tuj1 and Map2 co-stained positive cells in the Taxol 3.5 nmol/L and 5 nmol/L groups are higher, ^∗∗∗^*p* <0.001, and in Taxol 7 nmol/L group, ^∗^*p* <0.05. (e) The filament-like neurites of GFAP-positive glial cells derived from BNSCs are affected by the concentration of Taxol in the medium. With the increase in Taxol concentration, the filament-like neurites gradually elongate, presenting the characteristics of a “track-like” parallel arrangement.

We assessed the effects of Taxol on neurons during BNSCs differentiation. BNSCs could differentiate into Tuj1^+^ neurons and GFAP^+^ glial cells when the Taxol concentration in the differentiation medium was lower than 7 nmol/L. Furthermore, BNSCs showed a tendency to differentiate into neurons when the differentiation medium contained low-dose Taxol (lower than 7 nmol/L) (Figure 1(d)). Immunofluorescence showed an increased number of neurons and significantly larger axonal length in the Taxol-treated groups (Figure 2). Thus, the differentiation of BNSCs following Taxol intervention yielded more neurons (Figure 3(a)). Tuj1 and Map2 positively co-stained cells were found in all groups when the Taxol concentration in the differentiation medium was below 7 nmol/L. When the Taxol concentration was 3.5 nmol/L or 5 nmol/L, the ratio of Tuj1 and MAP2 co-stained positive cells was the highest (Figure 3(c), 3(d)).

We also detected the extension of neurons after Taxol treatment. In the control group without Taxol, the BNSCs differentiated into Tuj1-positive neurons and GFAP-positive glial cells, with the average extension of neuronal axons being 75.9 ± 4.8 *μ*m (Figure 3(b)). The axon length showed significant extension when Taxol concentration was below 7 nmol/L. When the Taxol concentration was 3.5 nmol/L and 5 nmol/L, the average extension was the highest, at 390.4 ± 5.5 *μ*m and 409.9 ± 10.5 *μ*m (Figure 3(b)), respectively, differing significantly relative to other Taxol-treated groups. When the Taxol concentration was 5 nmol/L, the axonal extension was the highest, and within the field of view, the extension reached up to 550 *μ*m for a single neuron (Figure 3(b), 3(c)). Taken together, if BNSCs were intervened using the microtubule stabilizer, Taxol, with an appropriate concentration during the differentiation, the obtained axons of functional neurons showed exponential extension.

During the differentiation of BNSCs into neurons and glial cells, the concentration of the microtubule stabilizer, Taxol, influenced the type and polarity of neuronal cells. When the Taxol concentration was below 3.5 nmol/L, Tuj1-positive cells were mostly pseudounipolar or bipolar neurons. When Taxol concentration was in the range of 3.5-7 nmol/L, Tuj1-positive cells were mainly multipolar neurons. When the Taxol concentration was lower than 10 nmol/L, with an increasing concentration, the filamentous protrusions of GFAP-stained positive glial cells gradually elongated and thickened and exhibited a parallel arrangement. Tuj1-positive axons extended along the cellular scaffold formed by the filamentous protrusions of parallel glial cells (Figure 3(e)).

### 3.4. HE Staining Shows the Success of the Rat T8-T9 Spinal Cord Transection Model

**Figure 4:**
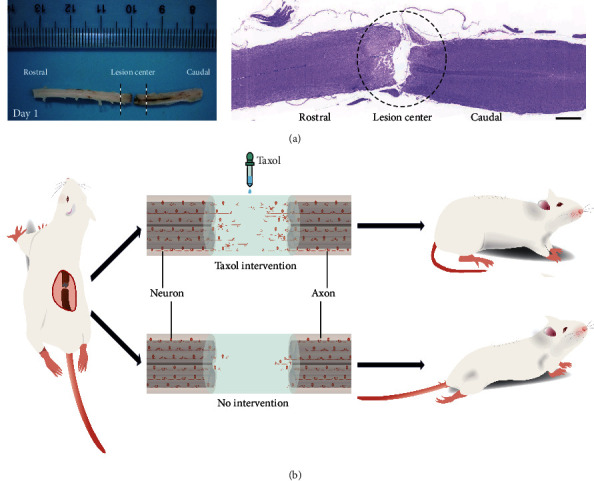
(a)The spinal cord tissue of T8-T9 spinal cord transection injury model rat is obtained on 1th day after operation, and it shows an obvious gap in T8-T9 spinal cord tissue. HE-staining shows an obvious gap in T8-T9 spinal cord, indicating successfully injured. Scale: 1000 *μ*m. (b) *In vivo* experiment diagram of low-dose Taxol can directly reflect the effect of low-dose Taxol on neuronal axon extension and limb motor functional recovery after SCI. The general idea of the diagram is that the addition of an appropriately low dose of Taxol combined with biomaterials could effectively increase the number of neurons and axonal extension, compared with no intervention group (with biomaterials), thus promoting the recovery of motor functions in rats after SCI.

We first established the rat SCI model with T8-T9 spinal cord transection and performed HE staining to verify the success of SCI. HE results showed an obvious gap in T8-T9 spinal cord, indicating successfully injuried (Figure 4(a)).

### 3.5. Taxol Promotes Motor Function Recovery and *β*III-Tubulin Expression in Injured Areas in Rats with SCI

*In vitro* experiments showed that low-dose Taxol could promote BNSCs differentiation into neurons and extend the axonal length. We further conducted *in vivo* experiments in rats to check whether low-dose Taxol was effective for the recovery of motor functions. The results show that the addition of the appropriately low-dose of Taxol to the SCI microenvironment in injured areas in rats could effectively increase the number of neurons and axonal length, thus promoting the recovery of motor functions in rats after SCI (Figure 4(b)).

**Figure 5:**
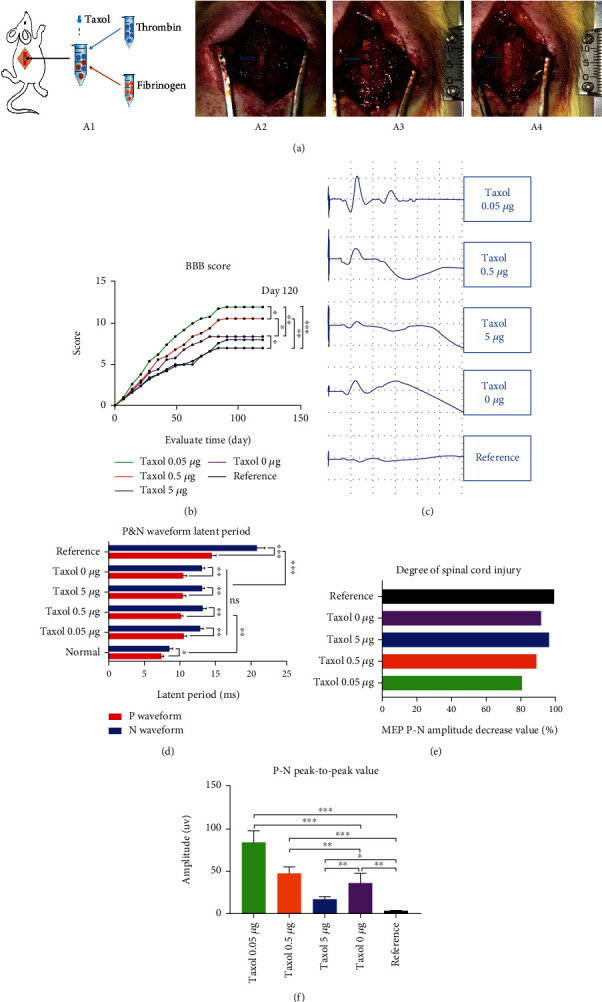
Low-dose Taxol promotes functional recovery in SCI rats. (a) ‘A1': Experimental flow chart. ‘A2': T8-T9 lamina is removed, and the spinal cord tissue below the lamina is exposed. The exposed spinal cord tissue is shown by the arrow. ‘A3': Spinal cord tissue with a length of 4 mm is removed in the transverse section to establish the acute stage model for rat SCI. ‘A4': Thrombin-fibrinogen materials carrying different doses of Taxol are implanted immediately into the lesion sites of the injured rats. (b) BBB scores for motor function in each group at 120 days after injury. ^∗^*p* <0.05, ^∗∗^*p* <0.01, ^∗∗∗^*p* <0.001. (c) Electrophysiological CMEP waveform. The stimulation intensity is 25 mV; the recording voltage is 1 mV/D; the recording current is 5 mS/D. (d) The latency of P and N peaks in the Taxol 0 nmol/L group and Taxol 0.05, 0.5, and 5 nmol/L groups show no significant differences between normal rats and injured rats. The latent period of peaks P and N represent the nerve conduction ability in the injured area, and the Taxol-treated groups show significant improvements. (e) The P-N peak-to-peak amplitude in each group decreases to more than 80% of the normal value, indicating a conduction block during evoked potential conduction. (f) The P-N peak-to-peak amplitude of the Taxol 0.05 nmol/L and 0.5 nmol/L groups show a more significant advantage as compared to the non-Taxol group.

We administered rats with acute SCI with low doses of Taxol to detect recovery of function (Figure 5(a)). Joint movement degree of the rats in different groups varied, at 1-2 weeks post-surgery, with animals in the 0.05 *μ*g/rat Taxol and 0.5 *μ*g/rat Taxol groups showing a significantly higher degree of hindlimb motor function recovery relative to the other groups. In particular, most rats in the 0.05 *μ*g/rat Taxol group showed extensive movement in hip and knee joints. Two weeks after surgery, the BBB score of rats in each group continued to increase. Two-to-eight weeks after surgery, the BBB score of rats in each group showed the most significant increase. The increase in the 0.05 *μ*g/rat Taxol and 0.5 *μ*g/rat Taxol groups was the most significant (Figure 5(b)), while that in the 5 *μ*g/rat Taxol group was negligible. In the 0 *μ*g/rat Taxol group the increase was moderate. Ninety days after surgery, the BBB score in each group suggested that the hindlimb motor function recovery of rats plateaued (Figure 5(b)). The baseline group entered the plateau phase the earliest. In this phase, extensive movements in the three joints of the hindlimb were found. There was no weight-bearing movement of the palmar surface of the paw or coordinated movements of the front and hind limbs. These hindlimb movements were considered to be entirely spontaneous; neurological functional recovery in the injury area was thus not achieved [17–19]. The BBB score in the 0.05 *μ*g/rat Taxol and 0.5 *μ*g/rat Taxol groups during the plateau phase was 11-13, which was significantly higher relative to other groups (Figure 5(b)). Some of the rats entering the plateau phase gradually changed foot dorsum-ground contact to the plantar surface-ground contact and recovered weight-bearing movement. A small number of rats showed frequent coordinated movements of the front and hind limbs.

We performed the CMEP and CSEP tests for each group of rats. The CMEP amplitude indicated the integrity of the functional status of the central motor pathway and directly reflected the repair condition after nervous system injury; no wave was detected in the CSEP test (Figure 5(c)). There were no significant differences in the latency of CMEP among all groups (Figure 5(d)). A decrease in the CMEP amplitude greater than 80% of the normal group suggested neurological damage symptoms and the difference was statistically significant (Figure 5(e)). The P-N peak-to-peak amplitude differed significantly between groups. The P-N peak-to-peak amplitude in the 0.05 *μ*g/rat Taxol group increased by a large margin, followed by the 0.5 *μ*g/rat Taxol and 0 *μ*g/rat Taxol groups, suggesting that Taxol in these groups also exerted a positive effect on post-injury repair (Figure 5(f)). In contrast, the P-N peak-to-peak amplitude in the 5 *μ*g/rat Taxol group was not significantly different, indicating no recovery in motor functions of rats in this group (Figure 5(f)). These electrophysiological findings indicate that the objective indices of *in vivo* electrophysiological detection and the subjective indices of the BBB score are consistent. Tissue materials containing 0.05-0.5 *μ*g/rat of Taxol promoted recovery of motor functions after SCI.

**Figure 6:**
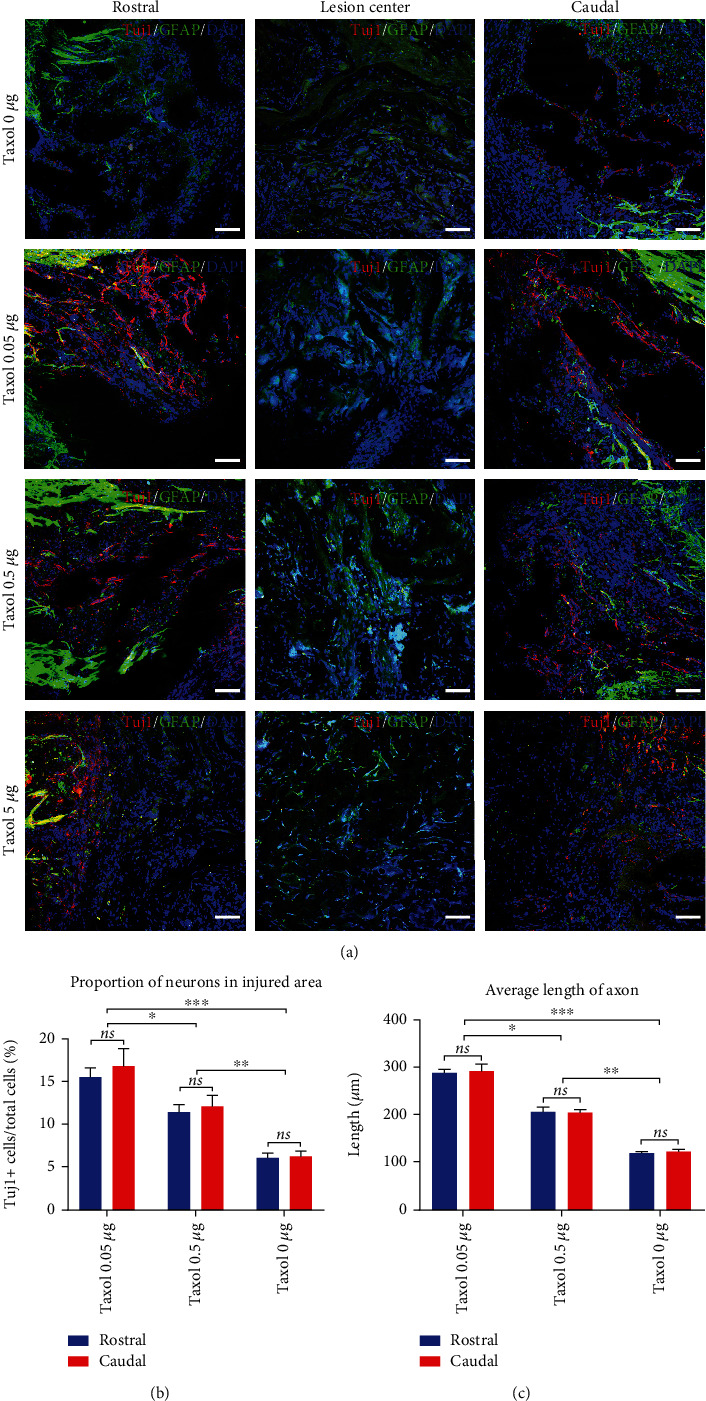
Tuj1^+^ cells in the injury areas following SCI after a low-dose Taxol treatment. (a) The low-dose Taxol group shows significant advantages as compared to the non-Taxol group in terms of the number of Tuj1-stained positive cells and axonal extension. Red, Tuj1; Green, GFAP; Blue, DAPI. Scale: 100 *μ*m. (b) Statistics for neurons in injury areas in the 3 groups. ^∗^*p* <0.05, ^∗∗^*p* <0.01, ^∗∗∗^*p* <0.001. (c) Statistics of axonal length in the injury areas in the 3 groups. ^∗^*p* <0.05, ^∗∗^*p* <0.01, ^∗∗∗^*p* <0.001.

Tissue samples from all study groups were assayed by immunofluorescence staining. Immunofluorescence images indicated that the number of neurons and axons increased significantly in the 0.5 *μ*g/rat and 0.05 *μ*g/rat Taxol groups (Figure 6(a)). We statistically analyzed the results of Tuj1^+^ cells in each group and found that the 0.05 *μ*g/rat Taxol group exhibited the highest number of Tuj1^+^ cells in the injury area, followed by the 0.5 *μ*g/rat Taxol group (Figure 6(b)). The 0 *μ*g/rat Taxol group had fewer Tuj1-positive cells (Figure 6(b)). The axons in the injury area showed good extension in the 0.05 *μ*g/rat group, with the longest axons among the three groups (Figure 6(c)). There were a few Tuj1-positive cells in the 5 *μ*g/rat Taxol group. Therefore, high-concentration Taxol inhibited the survival of neuronal cells in the injury area, thereby limiting the recovery of rat motor functions. The addition of an appropriately low dose of Taxol to the SCI microenvironment could effectively increase the number of neurons and axonal length, thus promoting the recovery of motor functions in rats after SCI.

## 4. Discussion

Microtubules in neurons are more stable than those in non-neuronal cells and effectively support cellular transport and maintain the normal structure of neurons [20]. After an injury, moderate microtubule stabilization prevents axonal retraction and the enlargement of the apical axon after injury which overcomes the growth inhibitory effects of myelin in the central nervous system [6]. The severed axons gradually retract to a spherical growth cone whose surface area increases over time for one month and the ratio of the stable detyrosinated microtubules to dynamic tyrosinated microtubules increases in the center of the growth cones [5]. Microtubules are disorganized and accumulated in the growth cones, whereby they establish and maintain neuronal polarity and regulate neuronal morphology, cell division, cell motility, and intracellular transport [21]. Axonal growth occurs mainly within the growth cones, and dynamic microtubules within the growth cones play an important role in the structural adjustment and growth of axons [22].

Microtubules are exceptionally sensitive to low temperatures, impact forces, and drugs, which cause instability and depolymerization [23]. Both cellular signaling pathways and exogenous drug interventions affect axonal growth through microtubules in the growth cones [3, 24]. Taxol affects the dynamic equilibrium between the dimer form of *α*-tubulin and *β*-tubulin and polymerized microtubules. Conversion of GTP/GDP is the main regulatory mechanism underlying the increase or decrease of microtubule stability [25]. Taxol promotes the assembly of tubulin into microtubules and stabilizes them by binding to GTP [26].

Although the total number of cells decreased after low-dose Taxol application, the number of neurons obtained in each group remained constant. Glial cells reduced following Taxol treatment, which may be due to the preference of NSCs differentiation toward neurons. In recent years, studies on spinal cord scar removal to promote spinal nerve regeneration have shown that a reduction in glial cells is conducive to the recovery of motor functions [27]. Therefore, the recovery of motor functions in rats *in vivo* is due to the combined effect of the increased neurons, extended axons, and decreased glial cells after the application of Taxol. The application of thrombin-fibrinogen materials, a replacement for spinal cord tissue, also plays a positive role.

Low-dose Taxol effectively extended axonal length, resulting in nearly 5-fold longer axons than those in the non-intervention state. Moreover, the Taxol concentration range may influence neuron type and contribute to the formation of more mature neurons at a higher concentration. We reasonably speculate that the moderate stabilization of microtubules during early neuronal polarization affected neuronal polarity and enhanced tubulin stability [28]. Our *in vivo* experiment also corroborated that low-dose Taxol treatment could promote the expression of *β*III-tubulin in the injured area and improve motor functions in rats in the acute phase of spinal cord transection. Taxol has been commonly used as a broad-spectrum antitumor drug for more than 20 years and its clinical safety is proven [29]. The discovery and evaluation of new pharmacological effects of Taxol can provide a good option for the clinical treatment of SCI.

## 5. Conclusions

Low-dose Taxol promoted efficient differentiation of NSCs to functional neurons; significantly extended neuronal axons *in vitro,* and promoted the expression of *β*III-tubulin in the injured area *in vivo*, thereby improving motor functions in rats in the acute phase of spinal cord transection. These findings are expected to advance the clinical application prospects of the microtubule stabilizer, Taxol, in the treatment of SCI.

## Data Availability

The data used to support the findings of this study are included within the article.
